# Repetition suppression and its contextual determinants in predictive coding

**DOI:** 10.1016/j.cortex.2015.11.024

**Published:** 2016-07

**Authors:** Ryszard Auksztulewicz, Karl Friston

**Affiliations:** Wellcome Trust Centre for Neuroimaging, Institute of Neurology, University College London, London, United Kingdom

**Keywords:** Repetition suppression, Predictive coding, Mismatch negativity, Perceptual inference, Perceptual learning

## Abstract

This paper presents a review of theoretical and empirical work on repetition suppression in the context of predictive coding. Predictive coding is a neurobiologically plausible scheme explaining how biological systems might perform perceptual inference and learning. From this perspective, repetition suppression is a manifestation of minimising prediction error through adaptive changes in predictions about the content and precision of sensory inputs. Simulations of artificial neural hierarchies provide a principled way of understanding how repetition suppression – at different time scales – can be explained in terms of inference and learning implemented under predictive coding. This formulation of repetition suppression is supported by results of numerous empirical studies of repetition suppression and its contextual determinants.

## 1 Introduction

The effect of stimulus repetition on neural responses is one of the most studied phenomena in neuroscience. Typically, repeated stimuli evoke neural activity with amplitudes smaller than responses to novel stimuli. Although repetition suppression is often portrayed as an expression of relatively simple mechanisms, such as neural fatigue (Grill-Spector, Henson, & Martin, 2006), its dependence on statistical regularities in the environment and other contextual factors casts repetition suppression as a consequence of sensory predictions (e.g., Summerfield, Trittschuh, Monti, Mesulam, & Egner, 2008). The predictive coding framework provides a principled explanation of repetition effects in terms of perceptual inference and learning, mediated by changes in synaptic efficacy (Friston, 2005). Adaptive changes in coupling of neuronal populations within areas and connectivity between areas are means of optimising a neuronal (generative) model of the external world to provide more accurate and precise predictions about sensory inputs. Thus, repetition suppression can be understood in terms of ‘explaining away’ sensory prediction errors.

In the following, we will review modelling and experimental work on repetition suppression in the setting of predictive coding. First, we introduce the predictive coding framework and portray neuronal message passing in terms of descending predictions, ascending prediction errors, and modulatory precision. We will then show how the predictive coding scheme can be mapped onto a canonical cortical microcircuit. The subsequent section will focus on explaining the dynamics of repetition suppression using simulations and computational modelling of empirical data. This will be followed by a review of empirical studies on repetition suppression and its context sensitivity, with a special focus on the crucial role of predictions and precision in modulating the effects of stimulus repetition.

## 2 Predictive coding

In order to maintain their integrity (e.g., homoeostasis), biological systems have to minimise the excursions or entropy of their interoceptive and exteroceptive states. Since entropy is the average of *surprise* (also known as surprisal or self-information) over time, biological systems should continually minimise their surprise about sensory states. Mathematically, this is equivalent to maximising the Bayesian evidence for their model of sensory inputs, also known as Bayesian filtering. Predictive coding (Friston, 2005, Mumford, 1992, Rao and Ballard, 1999) is a popular, neurobiologically plausible Bayesian filtering scheme that decomposes the optimisation of the agent's (neuronal) model of the world into two tractable components; namely (1) changes in expectations about the sensory inputs and (2) the computation of prediction errors that are needed to change expectations.

**Fig. 1:**
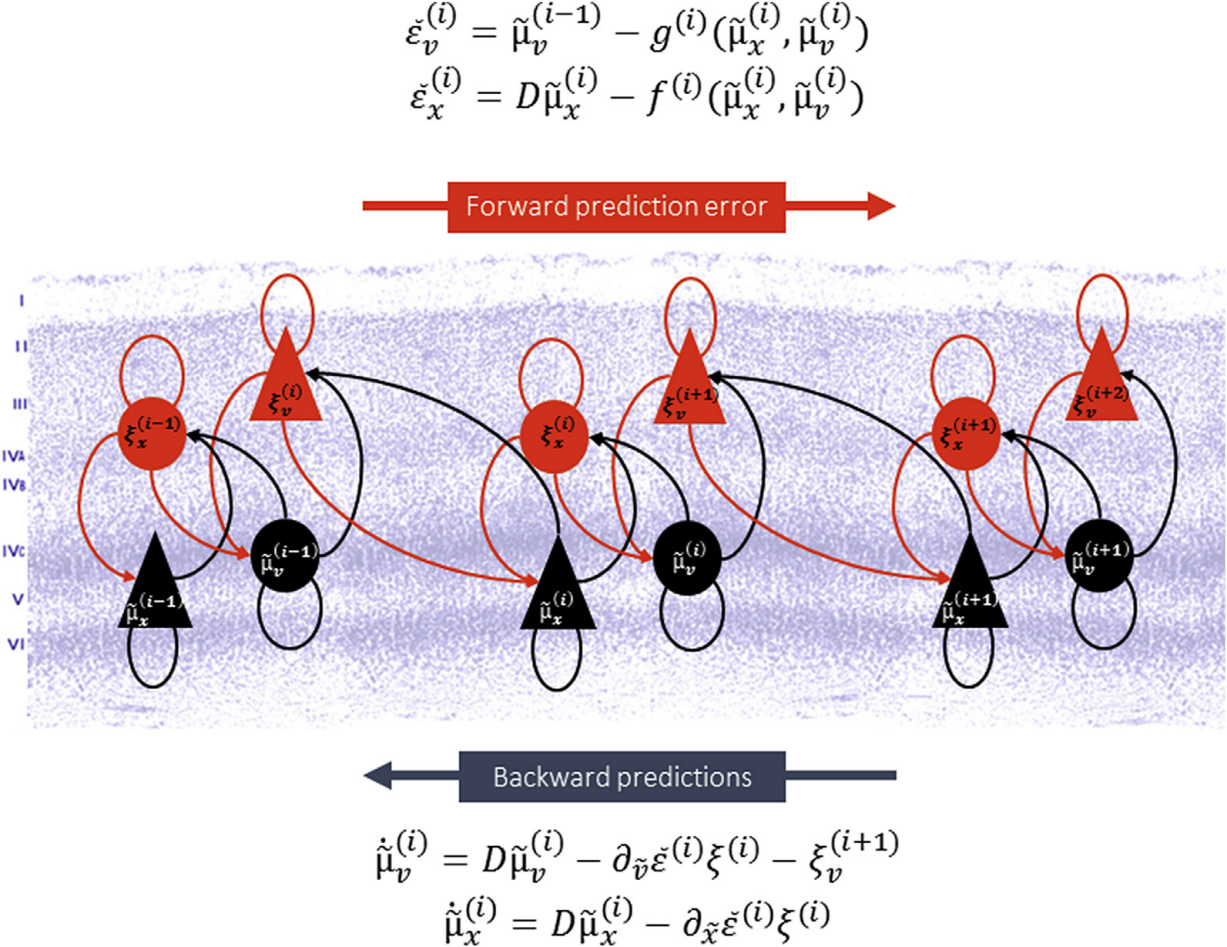
**Message passing in the predictive coding scheme**. This schematic illustrates the key asymmetry between ascending prediction errors and descending predictions in predictive coding schemes. The descending predictions serve to explain away sensory or neural input in lower areas. This is achieved by suppressing prediction errors, which in turn are used to optimise expectations about the (hidden) causes of sensory inputs. The equations describe the optimisation scheme that is discussed in more detail in the main text.

Minimising surprise – or maximising model evidence – lies at the heart of the free energy principle, where free energy provides a proxy for surprise that, under simplifying assumptions, can be reduced to prediction error (Friston, 2010). This means one can understand the process of perception as the resolution of prediction errors, by changing top-down predictions about the causes of sensory input (Fig. 1). Intuitively, the predictions descending along the processing (e.g., cortical) hierarchy are compared against sampled sensory inputs in sensory cortex (or expectations as intermediate hierarchical levels). The ensuing prediction errors are then passed up the hierarchy to optimise expectations and subsequent predictions. If the ascending input matches the descending prediction, the prediction error will be low – as exemplified by repetition suppression. If the predictions are inconsistent with the incoming input, the prediction error will be high – as illustrated by mismatch negativity (Garrido, Kilner, Stephan, & Friston, 2009). In the following, the notion of perception under predictive coding will be unpacked in the context of repetition suppression.

The ability of the brain to infer the causes of its sensations rests upon the presence of statistical structure or contingencies in the environment. These contingencies can be embodied within a generative model describing the hierarchical and dynamic statistics of the external world:

(1) $$D{\tilde{x}}^{\left(i\right)}=f^{\left(i\right)}\left({\tilde{x}}^{\left(i\right)},\;{\tilde{v}}^{\left(i\right)}\right)+{\tilde{\omega }}_{x}^{\left(i\right)}$$

(2) $${\tilde{v}}^{\left(i-1\right)}=g^{\left(i\right)}\left({\tilde{x}}^{\left(i\right)},{\tilde{v}}^{\left(i\right)}\right)+{\tilde{\omega }}_{v}^{\left(i\right)}\text{.}$$

In the equations above, *v* denote causes representing (hidden) causes (e.g., the bark of a dog), while *x* denote states of the world mediating the influence of that cause on sensory signals (e.g., the acoustic consequences of a dog barking). Because these dynamics follow stereotyped trajectories over time, they endow the model with memory. In equations above, tilde is used to augment the variables with their generalised coordinates of motion, i.e.,

(3) $$\tilde{x}=\left[x,x^{\prime },x^{\prime \prime },\ldots \right]\text{.}$$

Eq. (1) describes the motion of states *x*^(*i*)^ (at *i*-th hierarchical level) as a nonlinear function *f* of causes and states themselves. Here *D* is a block-matrix derivative operator, with identity matrices on its first leading-diagonal. Eq. (2) describes the motion of causes at a hierarchically lower level *i-1* as a nonlinear function *g* of hidden causes and states at the level above. Random fluctuations in hidden causes and states are denoted by ${\tilde{\omega }}_{v}^{\left(i\right)}$ and ${\tilde{\omega }}_{x}^{\left(i\right)}$ respectively.

Since the brain does not have direct access to the causes and states in the external world, it can only infer the most likely values under its generative model: mathematically, these values are expectations. In other words, the generative model maps from causes to sensory consequences, while perception solves the (usually very difficult) inverse problem which is to map from sensations to their underlying causes. An inversion of hierarchical dynamic models can be cast in terms of a hierarchical message passing scheme also known as predictive coding:

(4) $${\dot{\tilde{\mu }}}_{v}^{\left(i\right)}=D{\tilde{\mu }}_{v}^{\left(i\right)}-\partial_{\tilde{v}}{\tilde{\varepsilon }}^{\left(i\right)}\xi^{\left(i\right)}-\xi_{v}^{\left(i+1\right)}$$

(5) $${\dot{\tilde{\mu }}}_{x}^{\left(i\right)}=D{\tilde{\mu }}_{x}^{\left(i\right)}-\partial_{\tilde{x}}{\tilde{\varepsilon }}^{\left(i\right)}\xi^{\left(i\right)}$$

(6) $$\xi_{v}^{\left(i\right)}=\Pi_{v}^{\left(i\right)}{\tilde{\varepsilon }}_{v}^{\left(i\right)}=\Pi_{v}^{\left(i\right)}({\tilde{\mu }}_{v}^{\left(i-1\right)}-g^{\left(i\right)}({\tilde{\mu }}_{x}^{\left(i\right)},{\tilde{\mu }}_{v}^{\left(i\right)})$$

(7) $$\xi_{x}^{\left(i\right)}=\Pi_{x}^{\left(i\right)}{\tilde{\varepsilon }}_{x}^{\left(i\right)}=\Pi_{x}^{\left(i\right)}(D{\tilde{\mu }}_{x}^{\left(i\right)}-f^{\left(i\right)}({\tilde{\mu }}_{x}^{\left(i\right)},{\tilde{\mu }}_{v}^{\left(i\right)}))$$

This message passing suggests two distinct populations of neurons: one encoding the trajectory of the expectations (conditional means) of hidden causes ${\dot{\tilde{\mu }}}_{v}^{\left(i\right)}$ and states ${\dot{\tilde{\mu }}}_{x}^{\left(i\right)}$, which we can therefore label state-units, and one encoding the prediction errors ${\tilde{\varepsilon }}_{v}^{\left(i\right)}$ and ${\tilde{\varepsilon }}_{x}^{\left(i\right)}$ weighted by their respective precisions $\Pi_{v}^{\left(i\right)}\;$ and $\Pi_{x}^{\left(i\right)}$, which we can label error-units. These precisions are the inverse amplitude of the random fluctuations above, so that when the fluctuations are small, prediction errors become precise and are amplified. To simplify notation, $\partial_{\tilde{x}}$ and $\partial_{\tilde{v}}$ are used to denote a partial derivative with respect to hidden states and causes respectively. Temporal derivatives, e.g., $\partial_{t}x$, are denoted by a dot $\dot{x}$.

These equations may look complicated but are formally simple and quite revealing in terms of which (neuronal) units talk to each other. In brief, the equations suggest that error-units receive messages from populations in the same hierarchical level and the level above, while state-units are driven by error-units in the same level and the level below. The prediction errors from the same level $\xi^{\left(i+1\right)}$ and the level below $\xi^{\left(i\right)}$ provide lateral and bottom-up messages driving the conditional expectations ${\tilde{\mu }}^{\left(i\right)}$ towards better lateral and top-down predictions *f*^(*i*)^ and *g*^(*i*)^, serving to explain away the prediction error in the level below. Thus, predictions are subserved by the descending connections and prediction errors by ascending connections.

As mentioned above, prediction errors are weighted by their precision (or inverse variance). This allows for a dissociation of the magnitude of prediction error from its reliability – for example, in a noisy or volatile environment, continuous signalling of large prediction errors should not (necessarily) lead to large updates of expectations, since prediction errors will be very imprecise. Conversely, even minor deviations of sensory inputs from the descending predictions can lead to large updates of the conditional expectations if prediction errors are very precise. Crucially, precision itself has to minimise surprise (about the amplitude of prediction errors). Precision can therefore be manipulated exogenously; e.g., by changing the contrast or statistics (e.g., texture) of the stimulus, or endogenously; e.g., by attending to a particular sensory stream or changing the contextual expectancy of sequential stimuli. In other words, endogenous attention anticipates precise sensory information (or prediction errors). As we will see, the notion of precision will be key for interpreting some of the empirical findings showing that repetition suppression can be modulated by contextual factors such as attention.

## 3 Canonical microcircuits and predictive coding

**Fig. 2:**
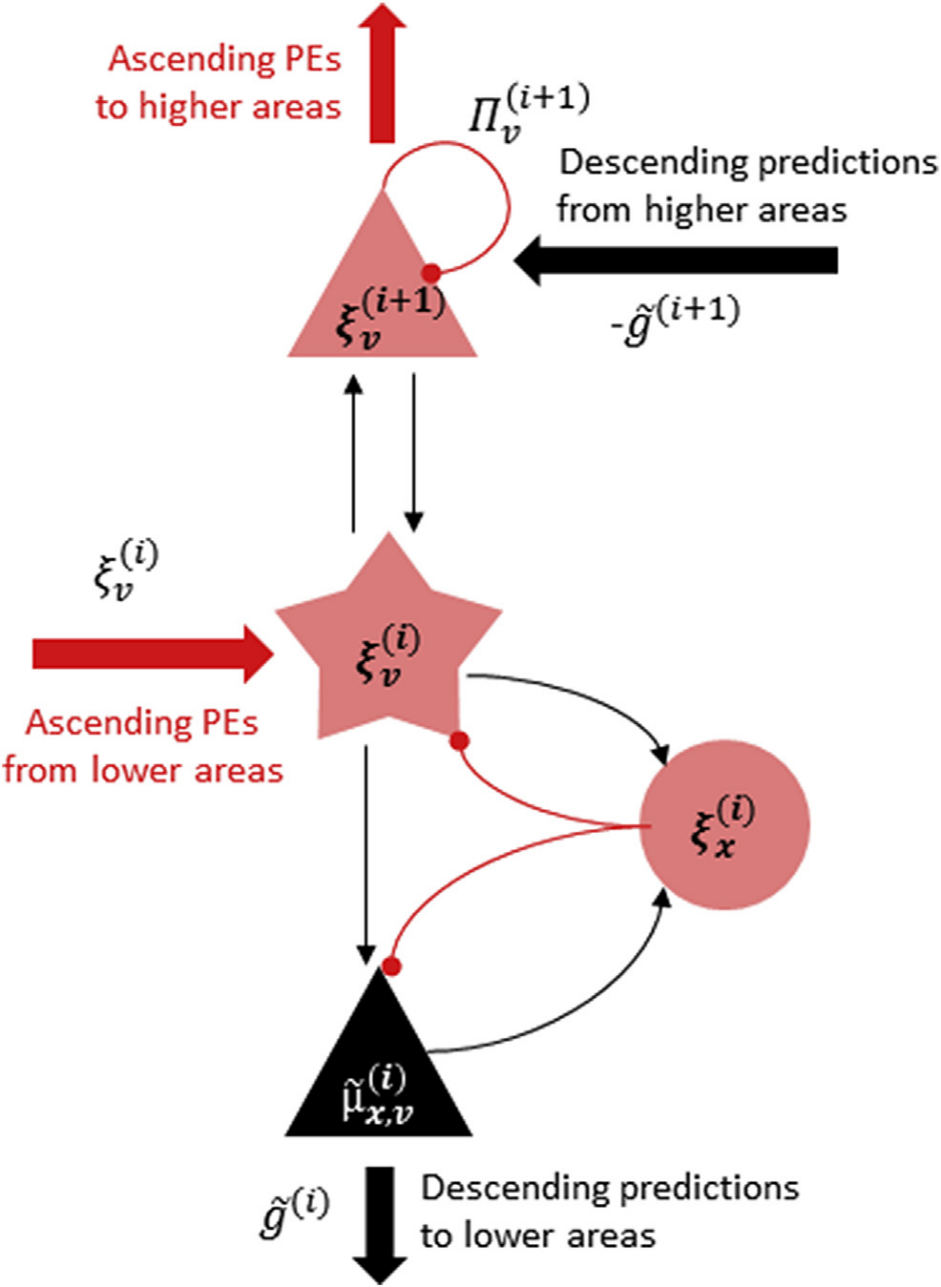
**Mapping the predictive coding scheme onto a canonical microcircuit**. A canonical cortical microcircuit has been proposed to implement predictive coding (Bastos et al., 2012). This schematic shows a speculative mapping of the key terms in the predictive coding scheme (predictions and prediction errors about hidden causes and states) onto distinct neuronal populations. Here, prediction errors about hidden causes from hierarchically lower areas are received by spiny stellate cells in the granular layer. The spiny stellate cells also receive inputs from inhibitory interneurons, encoding the prediction errors about hidden states (i.e., describing the dynamics at a given hierarchical level). These prediction errors are reconciled with descending predictions from hierarchically higher areas received by the superficial pyramidal cells, which reciprocate the ensuing prediction errors. At the same time, predictions are reconciled in the deep pyramidal layers and relayed to hierarchically lower areas.

The notion that the brain implements a predictive coding scheme in its cytoarchitecture (Mumford, 1992) has recently been put forward in the form of a canonical cortical microcircuit (Bastos et al., 2012) (Fig. 2). It draws upon the known laminar asymmetries of ascending and descending connections in the brain, with ascending connections (from hierarchically lower to higher regions) originating predominantly in superficial (supragranular) layers of the cortical sheet and targeting spiny stellate cells in the granular layer – and descending connections originating predominantly in deep (infragranular) layers and targeting all layers with the exception of the granular layer (Felleman & Van Essen, 1991). These asymmetries map neatly onto the distinction between predictions being propagated from hierarchically higher to lower regions in the message passing scheme and prediction errors being propagated in the opposite direction. Thus, in the canonical microcircuit for predictive coding, the deep pyramidal cells are associated with encoding predictions about the causes of inputs to a given area, while the superficial pyramidal cells are thought to represent prediction errors resulting from a comparison of top-down predictions with expectations in the microcircuit – or sensory input at the lowest hierarchical level.

Ascending and descending connections have also been shown to have distinct spectral profiles. Extensive work in the macaque visual system has demonstrated spectral asymmetries between superficial and deep cortical layers. Within cortical areas, activity in supragranular layers has been linked to local synchronisation of gamma-band oscillations, while neurons in infragranular layers typically synchronize in lower frequency bands such as alpha and beta (Buffalo et al., 2011, Roberts et al., 2013, Xing et al., 2012). Similarly, synchronisation between areas in the gamma band has been shown to subserve ascending connections from hierarchically lower to higher regions (Bosman et al., 2012), while descending connections are more likely mediated by inter-areal synchronisation in the beta frequency band (Bastos et al., 2015a, Bastos et al., 2015b) Interestingly, this spectral asymmetry between superficial and deep layers follows from a closer inspection of the mathematical form of the predictive coding scheme. Translating prediction errors into predictions rests on a linear accumulation of prediction errors to give slowly fluctuating estimates of hidden causes [Eqs. (4), (5)]. As such, translating prediction errors into predictions – or passing neuronal messages from superficial to deep cells – entails a loss of higher frequencies. Conversely, translating predictions into prediction errors rests upon a *nonlinear* function [Eqs. (6), (7)], which creates high-frequency prediction errors (imagine squaring a sine wave to double its frequency from beta to gamma). This raises the intriguing possibility that rather than constituting distinct physiological phenomena, different frequency bands form a spectrum determined by the form of the neuronal microcircuit. In other words, the laminar asymmetries inherent in microcircuits and hierarchical message passing require prediction error and prediction propagation to be mediated in different frequency bands.

Mapping the predictive coding scheme onto a canonical cortical microcircuit has important consequences. Since in the predictive coding scheme the descending predictions are subtracted from expectations in lower levels to form prediction errors, descending connections from deep pyramidal cells should inhibit activity in hierarchically lower areas (Murphy and Silito, 1987, Olsen et al., 2012, Silito et al., 1993), possibly using polysynaptic connections via inhibitory interneurons in Layer 1 (Chu et al., 2003, Meyer et al., 2011, Wozny and Williams, 2011). Similarly, the nonlinearity inherent in the generative model prescribing top-down predictions speaks to a modulatory character of descending predictions (Bullier et al., 1996, Covic and Sherman, 2011, De Pasquale and Sherman, 2011, Mignard and Malpeli, 1991, Sherman and Guillery, 1998). In the context of repetition suppression, the inhibitory effects of descending connections should attenuate the amplitude of neural responses, when expectations can be successfully predicted by hierarchically higher areas. The modulatory effects of descending connections, on the other hand, will manifest as changes in the precision of prediction errors; possibly at a slower time scale – such as changes in the attentional set or during perceptual learning. The dual role of descending connections (cf. Kanai, Komura, Shipp, & Friston, 2015) will be addressed in more detail in later sections.

## 4 Models of repetition suppression based on predictive coding

**Fig. 3:**
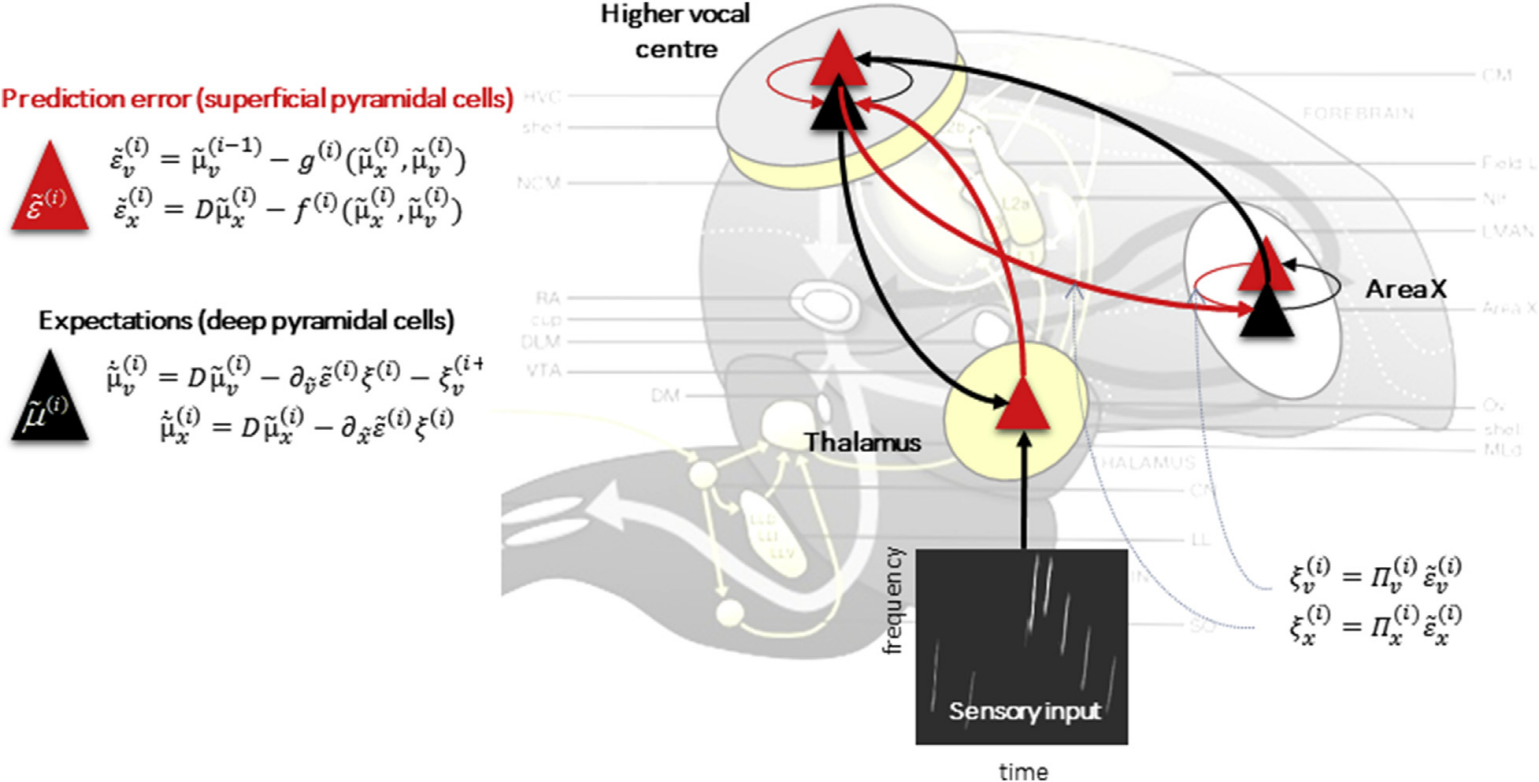
**Simulating brain-like dynamics to perform perceptual inference and categorisation**. The predictive coding scheme was used to simulate brain-like dynamics in an artificial avian brain (Friston & Kiebel, 2009). The goal of the agent was to perceive and categorise streams of auditory input, an example of which is shown in the lower inset. The activity in the thalamus, which receives the sensory input, is continually suppressed by descending predictions from hierarchically higher areas. Connections mediating prediction error propagation are shown in red, while descending connections subserving prediction propagation are shown in black.

Many known characteristics of repetition suppression emerge in simulations of predictive coding. This has been previously shown in a formal model of an artificial brain perceiving sequences of sensory events, simulated using attractor dynamics (Friston & Kiebel, 2009) (Fig. 3). In this model, the predictive coding scheme was employed to allow the agent to infer, categorize and learn its sensory inputs. As described above, the predictive coding scheme formalises the notion that the brain estimates hidden causes and states, representing the hierarchical structure and dynamics of the environment, using precision-weighted prediction errors. This can be mapped onto canonical cortical microcircuits whose superficial pyramidal cells encode prediction errors about hidden causes, propagated to hierarchically higher areas via ascending connections (Bastos et al., 2012). Similarly, deep pyramidal cells send predictions via descending connections to levels below.

Here, this architecture was used to model an artificial songbird, assuming a simplified functional anatomy of birdsong generation and perception. The artificial agent's goal was to categorize three trajectories of sensory inputs corresponding to three birdsongs. A predictive coding scheme was used to model perceptual inference and categorisation. In perceptual inference, after a few hundred milliseconds of listening to any of the three birdsongs, the simulated neural activity corresponding to descending predictions about sensory input recovers the trajectory of the input actually presented to the agent. In perceptual categorisation, the expectations of hidden causes (that controlled the trajectories) at a higher level disambiguated or categorised the three songs. This provides an example of how sequences of sensory events unfolding in time can be predicted and mapped to more abstract locations in perceptual space, using predictive coding.

**Fig. 4:**
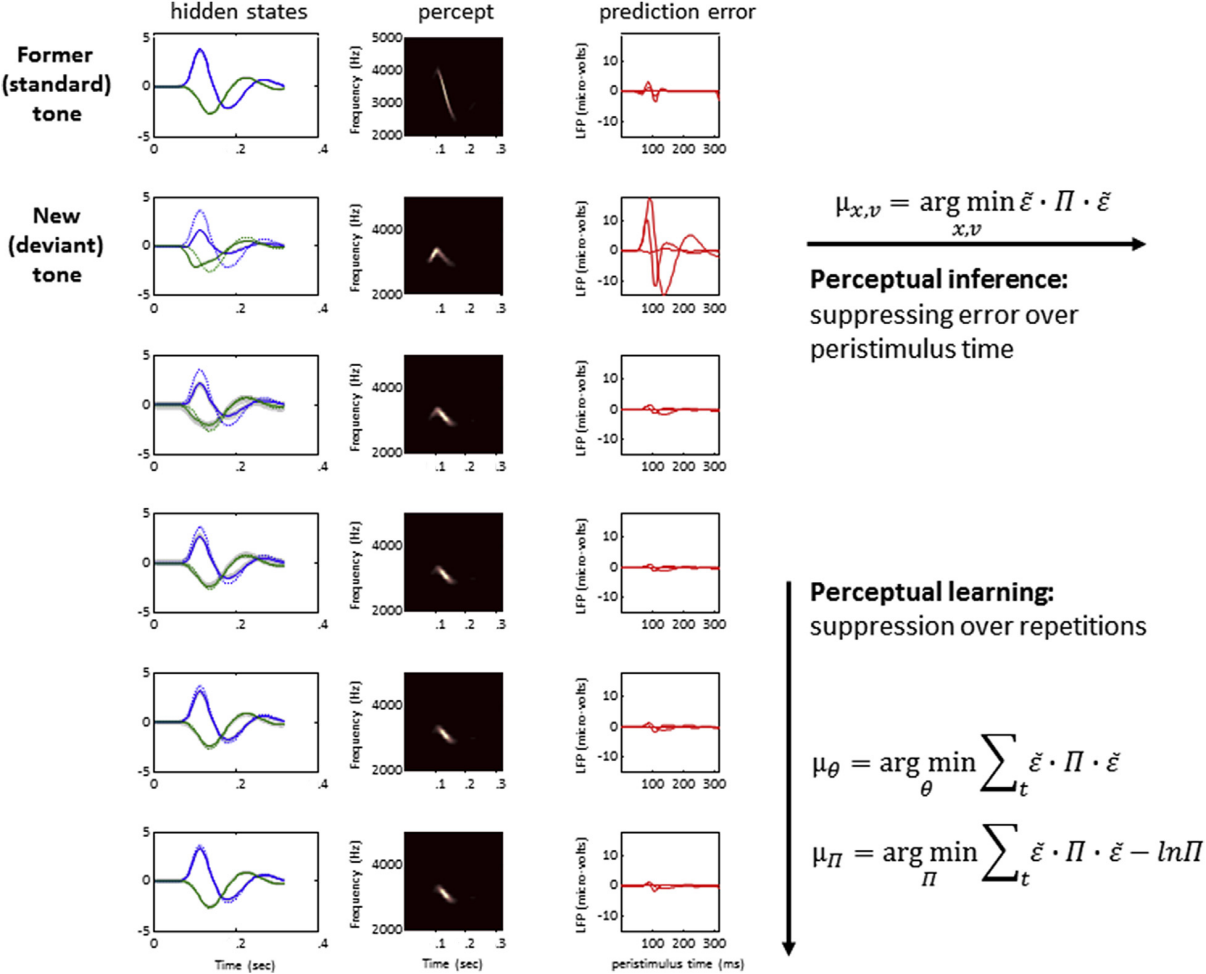
**Repetition suppression dynamics at separate time-scales**. The neural responses to repeated presentation of auditory stimuli, as simulated in the artificial avian brain (Friston & Kiebel, 2009), show repetition suppression effects at different time-scales. The left column contains panels illustrating the evolution of hidden states and causes at the lowest hierarchical level states (dotted: true states, solid: conditional expectations). They describe the frequency and amplitude of the sensory input, whose time-frequency representation is shown in the middle column. The right hand panels depict prediction error evolving over the course of each trial. The uppermost row corresponds to a standard stimulus, while in the second row a new stimulus is presented, resulting in a clear prediction error. Within trials, perceptual inference leads to a suppression of prediction error over peristimulus time. Across trials, perceptual learning (i.e., optimising the parameters and precision hyperparameters of the model) leads to repetition suppression.

Crucially, by simulating evoked responses to repeated chirps, the same model can reproduce the dynamics of repetition suppression – and, conversely, mismatch negativity – at different time-scales (Fig. 4). To investigate the responses of the artificial agent to repeated stimuli, a roving oddball paradigm was simulated. In this paradigm, tones are repeated a number of times, after which one or more of their attributes are changed and the resulting tone is repeated several times. The first tone in a sequence is defined as the sensory *deviant*, while its subsequent repetitions gradually become a sensory *standard*. Predictive coding of these sensory events (separately for each trial) revealed that the simulated prediction errors at the first (lowest) level peak at around 100 msec after stimulus onset, corresponding to the classical N1 component, while prediction errors at the second level peak at 150–250 msec after stimulus onset, mimicking the typical latency of mismatch negativity and consistent with its neural generators relying on hierarchically higher areas than those of the N1 component (Garrido, Kilner, Kiebel et al., 2009). Prediction errors at both levels are progressively eliminated over peristimulus time due to recurrent message passing and perceptual learning. In addition to minimising prediction error over time on every trial, the prediction error in response to a novel stimulus has a higher amplitude than prediction errors to stimuli repeated in subsequent trials. In terms of the difference waveform, the MMN is largest following the first presentation of a sensory deviant and vanishes after approximately two repetitions, while the amplitude of the N1 component fails to recover fully by the fifth repetition, consistent with empirical findings (Garrido, Kilner, Kiebel et al., 2009). This sort of simulation shows that the process of suppressing error can be decomposed into perceptual inference, occurring over peristimulus time, and perceptual learning, occurring over the course of several trials. These two aspects of error suppression can be formalised in terms of expectations about dynamic hidden states and slowly changing model parameters (including precision):

(8) $$\mu_{x,v}=\underset{x,v}{\text{arg min}}\tilde{\varepsilon }\cdot \Pi \cdot \tilde{\varepsilon }$$

(9) $$\mu_{\theta }=\underset{\theta }{\text{arg min}}\sum_{t}\tilde{\varepsilon }\cdot \Pi \cdot \tilde{\varepsilon }$$

(10) $$\mu_{\Pi }=\underset{\Pi }{\text{arg min}}\sum_{t}\tilde{\varepsilon }\cdot \Pi \cdot \tilde{\varepsilon }-ln\Pi$$

Here, perceptual inference – as described in earlier paragraphs – rests upon using precision-weighted prediction errors to drive changes in conditional expectations of hidden states and causes [Eq. 8]. In perceptual learning, the message passing scheme is optimised on a slower time-scale in that its parameters *θ* [Eq. (9)] and precision parameters (hyperparameters) *Π* [Eq. (10)] change as a function of precision-weighted prediction errors accumulated over trials. Learning the (hyper-) parameters of the model over the course of several trials can be linked to changes in synaptic efficacy and correspond to short-term plasticity.

An empirical study modelled the dynamics of repetition suppression using Dynamic Causal Modelling (DCM) of event-related potentials (ERPs) in humans (Garrido, Kilner, Kiebel et al., 2009). A roving oddball paradigm was again used, comprising a structured sequence of pure auditory tones with sporadically changing frequency. An incidental visual task was used to maintain an attentional set away from the auditory stimuli. An analysis of the ERPs revealed that after the third presentation of any given tone, the evoked responses to all subsequent tones differed only slightly, with no detectable differences after the fifth repetition. Therefore, a parametric DCM was adopted to examine the form of repetition-dependent connectivity changes during the first five stimulus presentations.

**Fig. 5:**
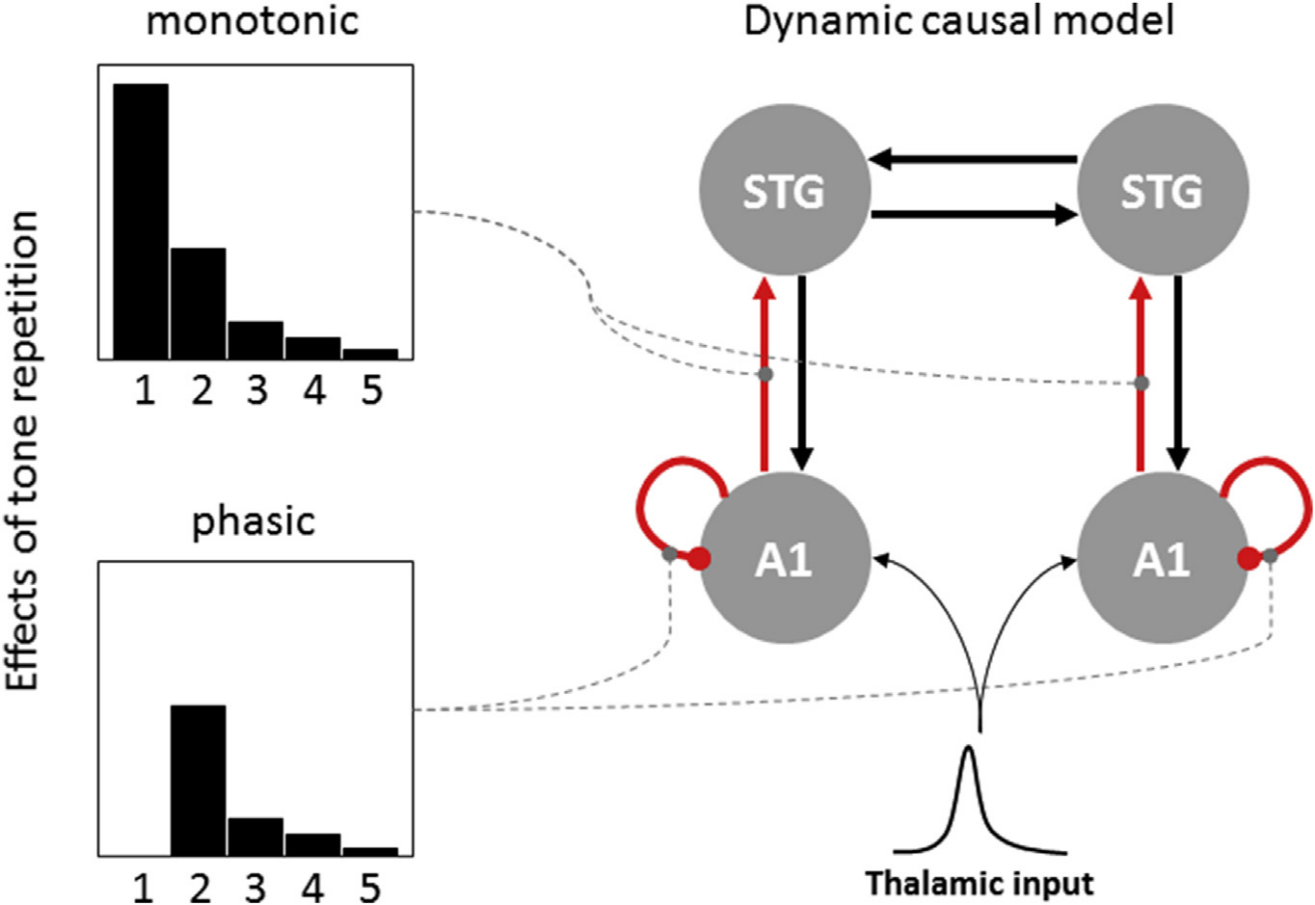
**Dynamics of adaptation and precision optimisation**. By applying Dynamic Causal Modelling to EEG data acquired in a roving oddball paradigm, Garrido, Kilner, Kiebel et al. (2009) provided evidence for distinct neuronal mechanisms underlying different time-scales of repetition suppression. The model that best explained the observed data suggests that intrinsic connections (modelling gain or precision effects) show biphasic plasticity effects, while ascending connections from lower (A1: primary auditory cortex) to higher (STG: superior temporal gyrus) auditory regions are modulated by a monotonic function of stimulus repetition. This is consistent with the notion that the expected precision of prediction error decreases after the presentation of a novel stimulus and recovers with its repetitions, while prediction error signalling is greatest for the first presentation of an (unpredicted) deviant stimulus and decreases with repetition.

In the DCM, the effect of stimulus repetition was decomposed into two time courses: a monotonic exponential decay function of stimulus repetition, mimicking slow cumulative effects, and a phasic (gamma-density) function peaking after the first repetition (Fig. 5). These two (basis) functions were used to model trial by trial changes in intrinsic connectivity, modelling the post-synaptic sensitivity of each source to all inputs, and/or extrinsic connectivity, modelling the post-synaptic sensitivity to source-specific inputs. The model that best explained the observed data featured a mixture of both effects, where biphasic plasticity (modelled as a combination of exponential decay and negative phasic functions) was deferred in intrinsic connections, while monotonic plasticity (modelled as exponential decay only) was linked to extrinsic (ascending) connections. This pattern of results is consistent with the notion that intrinsic connections reflect the precision (Friston, 2008), which should decrease after the presentation of a novel stimulus and recover with its subsequent repetitions (when more confident predictions can be made). On the other hand, a monotonic decrease in ascending connectivity speaks to an attenuation of prediction error signalling, after each subsequent repetition.

The two modelling approaches – one based on simulations of an artificial brain, one applied to empirical data – provide converging evidence for the notion that repetition suppression can be formulated in terms of predictive coding. With each stimulus repetition, the model of the sensory contingencies is gradually optimised, as encoded in changes to descending predictions. The descending predictions play a dual role, resolving the prediction error induced by sensory input and implementing changes in (expected) precision. Over the past few years, there have been further simulations of the mismatch negativity under predictive coding (e.g., Wacongne, Changeux, & Dehaene, 2012) and there have been many papers re-interpreting the mismatch negativity in terms of hierarchical inference in the brain (e.g., Winkler & Czigler, 2012). In the next section, the implicit mechanisms will be illustrated with examples taken from empirical studies of repetition suppression.

## 5 Empirical studies of repetition suppression in the context of predictive coding

### 5.1 The dual role of descending connections

Repetition suppression has been studied across species, imaging modalities, and stimulus categories. In the cat auditory cortex, single neuron recordings have shown a decreased response to standard stimuli and increased response to deviant stimuli within oddball paradigms (Ulanovsky, Las, & Nelken, 2003). These effects likely extend beyond auditory cortex to subcortical areas, with decreased responses to repeated stimuli reported in single-neuron recordings from rat inferior colliculus (Ayala & Malmierca, 2013) and the medial geniculate body of the thalamus (Antunes, Nelken, Covey, & Malmierca, 2010). Repetition suppression of subcortical activity is modulated, although not abolished, after deactivation of auditory cortex (Anderson and Malmierca, 2013, Antunes and Malmierca, 2011) as well as after pharmacological manipulation of GABA_A_ receptors in the inferior colliculus (Pérez-González & Malmierca, 2012) and medial geniculate body (Duque, Malmierca, & Caspary, 2014). This is consistent with the implementation of predictive coding within a canonical microcircuit (Bastos et al., 2012), where descending predictions are typically modelled as targeting inhibitory interneurons that enable superficial pyramidal cells to compare incoming sensory input with the descending predictions. As mentioned above, in the cortex this computational principle is thought to rely on descending connections terminating predominantly in layer 1, containing exclusively inhibitory interneurons. Similar mechanisms might be in place in subcortical areas. For example, descending cortico-thalamic projections target inhibitory cells in the reticular nucleus of the thalamus (Zhang & Jones, 2004). In the inferior colliculus, GABAergic neurotransmission has been shown to play a dominant role in shaping and modulating functionally specialised responses (Faingold, 2002). The findings that deactivating descending connections has a predominantly modulatory effect suggests that subcortical areas may receive descending predictions of the precision of ascending auditory information, contextualising this input to mediate temporal attention (Nobre, Correa, & Coull, 2007). Indeed, manipulating temporal regularity in the roving oddball paradigm leads to increased repetition suppression effects under predictable stimulus timing (Costa-Faidella, Baldeweg, Grimm, & Escera, 2011).

The evidence for a modulatory role of descending connections in repetition suppression of BOLD responses was further addressed in a DCM study using fMRI (Ewbank et al. 2011). Here, participants viewed repeated images of human bodies, which were either identical across repetitions or differed with respect to the size and/or view of the depicted body. BOLD responses were modelled in DCMs comprising regions of interest in the ventral visual stream – the extrastriate body area and the fusiform body area. Repetition suppression was modelled as modulating the extrinsic connections between the extrastriate and the fusiform regions (ascending, descending, both, or neither) and/or the intrinsic connections describing the self-inhibition of the extrastriate and the fusiform regions. Model comparison revealed that when images were presented under changing size or view conditions, stimulus repetition affected the descending connections from the fusiform to the extrastriate body area, as well as intrinsic connections in both regions. However, under constant size and view conditions, stimulus repetition also affected the ascending connections, reducing the strength of these connections. The change in descending connectivity with repetition suppression is consistent with perceptual learning that enables them to suppress ascending sensory input. Concurrent modulation of intrinsic connections corroborates the dual role of descending predictions not only in resolving prediction errors but also in modulating their precision. Finally, the additional decrease in strength of ascending connections by repetition of identical images suggests that neuronal populations in the extrastriate body area learn to predict low-level visual features, thereby attenuating prediction errors propagated from that area.

### 5.2 Precision modulation and repetition suppression

**Fig. 6:**
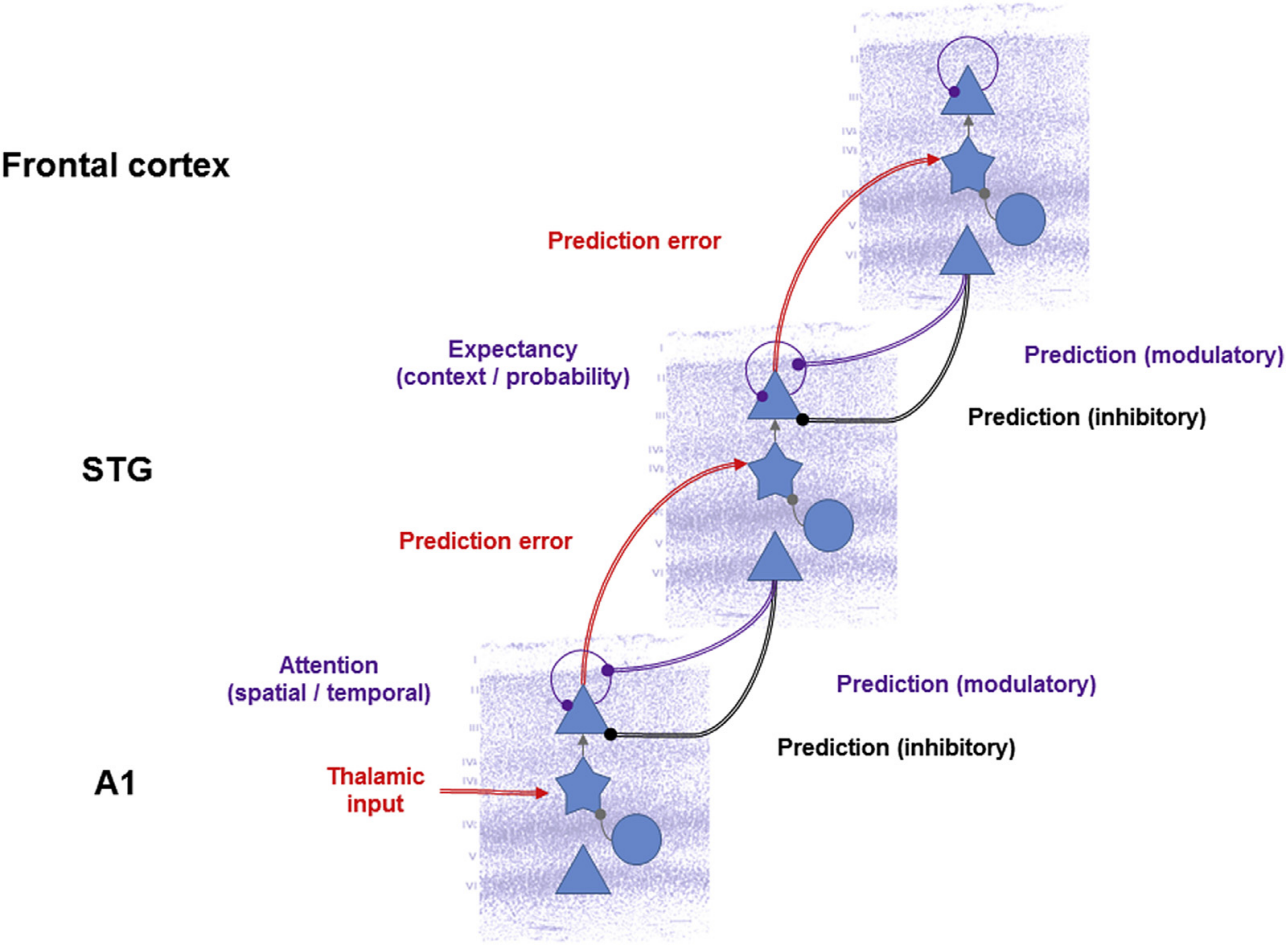
**Contextual factors and hierarchical message passing**. This schematic shows a tentative mapping of contextual factors modulating repetition suppression effects onto distinct levels of a cortical hierarchy. Attention acts as sensory gain control by modulating the precision of prediction errors in early sensory regions (Feldman & Friston, 2010). The disinhibitory effects of attention augment the differences between responses to sensory deviants and standards (Auksztulewicz & Friston, 2015). Contextual expectancy, manipulating by e.g., changing the probability of stimulus repetition in a longer sequence, is mediated by changes in precision higher in the hierarchy. This is consistent with the longer latencies of responses affected by manipulating repetition probability (Todorovic & de Lange, 2012) relative to the effects of repetition itself.

Since prediction errors are weighted by their expected precision, changing precision at various levels of the cortical hierarchy should modulate repetition suppression effects. Synaptic gain control mechanisms such as N-methyl-d-aspartate (NMDA), as well as classical neuromodulators such as acetylcholine (ACh), dopamine and serotonin are natural candidates to implement precision or gain control in prediction error signalling (cf. e.g., Iglesias et al., 2013). Thus, pharmacological manipulations of neurotransmission and neuromodulation should influence repetition suppression. Similarly, manipulating sensory precision by stimulus construction (e.g., increasing its signal-to-noise ratio) or task instruction (e.g., by altering attentional set) should modulate the effects of stimulus repetition. Furthermore, due to the nonlinearity inherent in precision-weighting of prediction errors, manipulating precision at higher hierarchical levels of abstraction or encoding, e.g., by changing stimulus expectancy or environmental volatility, should have different effects on repetition suppression related to manipulating precision at lower (sensory) levels (Fig. 6). In the following paragraphs, we will review studies providing evidence for the modulatory effects of changing the precision of sensory information at various hierarchical levels.

In predictive coding, the learning of statistical regularities in the environment rests upon optimising descending predictions (of content and context). This learning implicates short-term synaptic plasticity and neuromodulatory effects of the sort mediated by NMDA-type glutamate (for perceptual learning of content) and ACh receptors (for mediating attention through expected precision). Prediction errors, on the other hand, are conveyed by ascending (driving) connections that use fast AMPA-type glutamate receptors. This asymmetry is not only supported by empirical differences between ascending and descending projections but can be deduced from the form of Eqs. (4), (6): note that expectations are driven by a linear mixture of ascending prediction errors, while prediction errors depend upon non-linear functions of descending expectations (Friston, 2005). Thus, pharmacological manipulations of NMDA- and ACh-dependent processing should primarily influence learning and attention, leaving inference *per se* relatively intact. In the context of repetition suppression, impaired learning would be evident in a loss of the attenuation of evoked responses with stimulus repetition, while intact inference would manifest itself in a preserved response to sensory deviants. Exactly this pattern of results was observed in a double-blind study in which ACh receptor agonist nicotine (Baldeweg, Wong, & Stephan, 2006) was administered in a roving oddball paradigm. Drug administration, relative to placebo, increased the amplitude of neural responses in frontal areas to repeated stimuli, while leaving the negative components of event-related potentials in response to sensory deviants intact.

Similarly, a DCM study investigated the effects of galantamine (a cholinesterase inhibitor, increasing the availability of ACh in synapses) on mismatch negativity (Moran et al., 2013). An analysis of evoked responses to subsequent stimulus repetitions showed diminished repetition suppression to oddball stimuli in sensory cortices. Under placebo, as shown in a previous study (Garrido, Kilner, Kiebel et al., 2009), the expected precision of prediction errors is thought to be suppressed after the first occurrence of a stimulus and gradually increase with successive stimulus repetitions. This effect can be seen in Fig. 5, where precision recovers with repetitions of the new standard; thereby enhancing prediction error responses that are progressively attenuated by learning. The implication here is that repetition effects on learning and precision produce repetition suppression and enhancement respectively, where suppression would normally supervene. However, increasing the availability of ACh by galantamine administration should counteract any initial decrease of precision and augment repetition enhancement, leading to attenuated and delayed repetition suppression. Importantly, the DCM used to model this attenuation of repetition suppression empirically was based on a canonical microcircuit (Bastos et al., 2012). Cholinergic manipulation was linked to changes in gain (self-inhibition) of superficial pyramidal cells, consistent with its role in mediating the expected precision of prediction errors.

Another DCM study investigated the mechanisms underlying the effects of NMDA-receptor antagonist ketamine, administered in a roving oddball paradigm (Schmidt et al., 2013). Besides replicating previous findings (Garrido, Kilner, Kiebel et al., 2009) in terms of the winning model – which included a modulation of both intrinsic and extrinsic connections by sensory deviance – this study showed that ketamine reduced synaptic plasticity (extrinsic connections) but not adaptation (intrinsic connections) in the auditory cortex. This is consistent with a role of NMDA receptors in perceptual learning (that changes extrinsic connections), as distinct from a role of neuromodulation in precision or gain control (that changes intrinsic connections). Furthermore, changes in extrinsic connections were correlated with ratings of cognitive impairment induced by ketamine. These findings suggest that blocking the NMDA-type glutamate receptors impairs the ability to learn the (changing) parameters of a generative model efficiently. On the other hand, adaptive control of precision in the sensory cortex might rely more on classical neuromodulators, such as ACh. A more direct assessment (e.g., using optogenetic methods) of the relative contribution of different neuromodulatory mechanisms to hierarchical message passing remains an important subject of future studies.

Beyond pharmacological manipulations, sensory precision can be modulated by various exogenous and endogenous factors. Decreasing stimulus visibility, for instance, has been shown to abolish repetition suppression effects (Turk-Browne, Yi, Leber, & Chun, 2007), consistent with modelling work linking visual contrast to sensory precision (Brown & Friston, 2012). Similarly, directing attention to stimuli, which in previous modelling work had been linked to increased sensory precision (Feldman & Friston, 2010), has been shown to increase the amplitude of a mismatch response by disinhibiting early sensory areas (Auksztulewicz & Friston, 2015). In the latter study, DCM was used to model MEG data acquired from healthy participants performing a task in which sensory expectation and temporal attention were orthogonally manipulated. Specifically, auditory tones formed a roving oddball sequence, where changing tone frequency differentiated between sensory standards and deviants. Furthermore, attention could be deployed to (or diverted away from) different time windows in which the auditory tones could appear. Temporal attention was shown to significantly modulate sensory expectations, with the amplitude of evoked neural responses in auditory areas differentiating between sensory standards and deviants only when the tones were attended. Crucially, DCM revealed that the effect of attention was mediated by changes in descending connectivity from higher to lower auditory regions, targeting inhibitory interneurons in primary auditory cortex. This finding was consistent with previous fMRI studies in the visual domain, showing that repetition suppression of BOLD responses is modulated by spatial attention (Eger et al., 2004, Henson and Mouchlianitis, 2007) as well as feature-based attention (Moore et al., 2013, Yi and Chun, 2005, Yi et al., 2006). Although some forms of repetition suppression or mismatch responses can be preserved in the absence of attention (cf. Sussman, Chen, Sussman-Fort, & Dinces, 2014), the studies reviewed here suggest that attention – by increasing the gain at early processing stages, or the precision of sensory prediction errors – will serve as a modulatory factor, influencing the prediction error responses underlying repetition suppression.

Attention has often been operationalised as the relevance of a particular stimulus for the task at hand (Summerfield & Egner, 2009). A direct comparison of neural responses to relevant and irrelevant repeated stimuli provides evidence for the modulatory character of stimulus relevance, with respect to repetition suppression (Miller, Erickson, & Desimone, 1996), although these effects might not be universal across the cortex (Miller & Desimone, 1994). In these studies, macaques performed a variant of a delayed match-to-sample task in which the sample and target were separated by repeating non-match stimuli (e.g., ABB′A′, where A is the sample, A′ is the matching target, and repeating non-match BB′ stimuli separate the two). The macaques were trained to respond to the target stimulus only. Neurons in the prefrontal cortex that showed a suppression of their firing rate in response to repeated (task relevant) stimuli, relative to their first presentation in a trial (i.e., A′–A), also suppressed their responses to irrelevant repeated stimuli (B′), although to a lesser degree than for relevant repeated stimuli (Miller et al., 1996). In the inferior temporal cortex, on the other hand, the degree of suppression was not significantly modulated by task relevance – at least when compared to the sample stimulus A (Miller & Desimone, 1994). In both areas, however, neurons that showed repetition suppression of target-evoked activity fired most vigorously in response to the first occurrence of the non-match stimulus (B), indicating a strong prediction error response. Since neither of these studies analysed the interaction of repetition suppression and task relevance directly (A′–A vs B′–B), but only compared the firing rate of neurons in response to repeated stimuli relative to the sample (A′–A vs B′–A), further work is needed to directly assess the interactive effects of relevance and repetition on the firing rate of single neurons in areas beyond sensory cortex. Crucially, however, if task relevance provides a good operationalisation of attention, its modulatory effects should be most pronounced in early sensory areas (cf. Auksztulewicz & Friston, 2015).

In contrast to attention, which has been be linked to low-level (sensory) precision, manipulating precision at higher contextual levels has been addressed by manipulating repetition probability – building upon a distinction between repetition suppression and expectation suppression. The expectations here rely upon context (e.g., sequential structure or stimulus probability), and thus are presumably located at high hierarchical levels that can represent contextual factors. According to predictive coding, descending predictions of incoming repeated stimulus should suppress the prediction error resulting from the presentation of this stimulus. However, if the stimulus sequence is constructed such that stimulus alternation is more likely than repetition, presenting an identical stimulus twice should result in a prediction error, while valid expectations of a different stimulus should suppress prediction error. The initial finding that stimulus repetition probability affects repetition suppression (Summerfield et al., 2008) was based on fMRI data acquired from humans, and has since been replicated by other groups (Andics et al., 2013, Grotheer and Kovács, 2014, Larsson and Smith, 2012, Mayrhauser et al., 2014). The latter study (Grotheer & Kovács, 2014) also showed that the influence of expectation on repetition suppression depends on prior experience. Although repetition suppression occurred for both familiar (upright Roman letters) and unfamiliar (false fonts) stimuli, the influence of expectation on repetition suppression was only observed in the case of familiar stimuli. This suggests that extensive prior experience facilitates the forming of predictions which, at the contextual level, influence repetition suppression.

Interestingly, the effects of repetition suppression and expectation suppression have been shown to be dissociated in time using MEG (Todorovic & de Lange, 2012). While repetition suppression was most prominent at relatively early latencies (40–60 msec) of the auditory evoked response, expectation suppression was present at later latencies (100–200 msec), within the mismatch negativity range. The relatively early onset of repetition effects suggests that repetition suppression may reflect low-level expectations based on local transition probabilities (Garrido et al., 2007, Kiebel et al., 2008, Wacongne et al., 2011), and replicates previous findings showing that deviance magnitude (i.e., the absolute frequency of a deviant in the stimulus sequence) affects early components of the evoked response up to the N1 component (Horváth et al., 2008) but not the later components of the MMN proper. The effects of stimulus probability, on the other hand, might rely on hierarchically higher expectations about the sequence structure or likelihood of stimuli, induced by learning of the statistical regularities of the sequence. This pattern of results is consistent with a cascade of prediction errors that update predictions at progressively higher levels of the processing hierarchy, as reflected in the hierarchically distinct generators of the early and late components of the evoked response (Friston and Kiebel, 2009, Garrido et al., 2009b). Interestingly, at even later latencies (200–500 msec) repetition and stimulus probability showed an interaction effect, replicating the modulatory effects of expectation observed in fMRI.

As discussed above, repetition suppression is subject to attentional modulation. Similarly, the interaction of expectation and repetition has been shown to depend on top-down attention (Larsson & Smith, 2012). When participants attended to the visual stimuli, expectation influenced repetition suppression of BOLD activity in extrastriate areas. However, when participants diverted their attention away from the stimuli, only repetition suppression was observed (albeit the overall amplitude of the visual response was attenuated), while the effects of stimulus expectation were abolished. This finding might explain why the interaction of expectation and repetition was not replicated in an invasive study in the macaque inferior temporal cortex (Kaliukhovich & Vogels, 2011), since in that study stimuli were presented under passive fixation.

The results of studies investigating the interactions between repetition and attention are in line with the nonlinear, modulatory nature of precision or gain control in predictive coding. Due to this nonlinearity, independent manipulations of precision at different hierarchical levels might even have antagonistic effects on neural responses in sensory areas (Kok, Rahnev, Jehee, Lau, & de Lange, 2012). Therefore, we now turn to experiments showing that, under specific conditions, stimulus repetition might lead to enhanced neural activity.

### 5.3 Repetition suppression and repetition enhancement

Besides repetition suppression effects, several studies have reported repetition enhancement effects, or increased neuronal responses to repeated stimuli (for a review, cf. Segaert, Weber, de Lange, Petersson, & Hagoort, 2013). In a roving oddball paradigm administered in MEG (Recasens, Leung, Grimm, Nowak, & Escera, 2014), repetition enhancement has been shown to occur later (at 230–270 msec) than the repetition suppression effects (95–150 msec) which contribute to the mismatch negativity. Both of these effects could be localised to sources in auditory cortex including Heschl's gyrus and superior temporal gyrus, as well as middle temporal gyrus. However, repetition enhancement effects were associated with additional sources in the anterior insula. Functional dissociations between repetition suppression and enhancement have also been observed using fMRI for distinct stimulus categories. For example, repetition suppression characterised BOLD responses to familiar faces, while repetition enhancement was observed in response to unfamiliar faces (Henson, Shallice, & Dolan, 2000). Similarly, repetition suppression was reported under conditions of high stimulus visibility, while degrading stimulus visibility yielded repetition enhancement effects (Turk-Browne et al., 2007).

A recent fMRI study (Müller, Strumpf, Scholz, Baier, & Melloni, 2013) tried to reconcile these two apparently opposing phenomena. In this paradigm, novel visual scenes were presented to participants at low contrast and exposure duration (50 msec) a number of times. BOLD responses in scene-selective regions followed an inverted U-shape function of stimulus repetition, with the first five presentations of a novel stimulus showing gradual repetition enhancement and further presentations showing gradual repetition suppression. This suggests that while learning a new model of a stimulus, repetition leads to a gradual increase in the precision of perceptual predictions at higher levels in the hierarchy, consistent with the relatively late latency of repetition enhancement observed in MEG (Recasens et al., 2014). After the perceptual representation of a stimulus has been established, precision control can be deployed at lower levels in the sensory processing hierarchy, leading to (early-latency) repetition suppression. This deployment can be understood as a reduction of model complexity at the lower levels of the hierarchy. Formally, this may correspond to Bayesian model averaging, in which the predictions of different models are weighted according to their evidence. The ensuing Bayesian model average provides an optimal model, under which learning can proceed (FitzGerald, Dolan, & Friston, 2014).

Similar arguments have been raised in interpreting the results of another study (Zago et al., 2005), where the amplitude of BOLD repetition suppression – as well as behavioural performance (cf. Miyoshi et al., 2015) – was shown to depend on the exposure duration of the first stimulus in a non-linear fashion. At the shortest exposure duration examined (40 msec), BOLD response to subsequently repeated stimuli was not significantly suppressed, and in some areas was nominally enhanced (cf. Müller et al., 2013, although note that the two studies differ in the level of visual contrast and familiarity with the stimuli). For exposure durations up to 250 msec, the BOLD activity in extrastriate regions was progressively more suppressed in response to repeated stimuli. However, for even longer exposure durations (up to 1900 msec), the degree of repetition suppression decreased again, although it remained stable across a range of durations tested. This is consistent with the idea that at very short durations (e.g., 40 msec), the process of perceptual inference might be incomplete, recovering the hidden causes of sensory inputs with low precision – and resulting in weak repetition suppression. However, with longer exposure times of 250 msec, the hidden causes can be recovered with high precision, leading to stronger repetition suppression. Finally, longer exposure times can lead to further model optimisation and deployment of precision control to hierarchically lower areas, resulting in a steady level of repetition suppression.

Another attempt at integrating repetition suppression and enhancement effects provided evidence for their co-occurrence within extrastriate visual regions (de Gardelle, Waszczuk, Egner, & Summerfield, 2013). Based on fMRI data acquired in a paradigm in which face images were repeatedly presented to participants, two subpopulations of voxels were isolated in the fusiform face area (as well as at more posterior occipital sites in Brodmann Areas 18 and 19), showing repetition suppression and enhancement effects respectively. The two subpopulations in the fusiform face area were spatially clustered (for example, the right fusiform face area showed an anterior-posterior gradient of voxels showing enhancement vs. repetition), stable across experimental sessions, and showed different peak latencies of the BOLD response (again, with the repetition suppression effects occurring earlier than repetition enhancement) as well as differential functional connectivity patterns. Specifically, the activity of voxels showing repetition suppression correlated preferentially with activity in lower visual regions, suggesting that these voxels might be more responsive to ascending input. This is consistent with the findings of invasive studies in the macaque cortex, where inferior temporal cortex has proportionally more neurons showing repetition suppression (Miller & Desimone, 1994), in contrast to the prefrontal cortex with more neurons showing repetition enhancement (Miller et al., 1996). In terms of predictive coding, this suggests that voxels showing repetition suppression effects might be mediated by neurons signalling prediction errors, which become progressively weaker as stimulus repetition leads to their more effective reconciliation with the descending predictions. On the other hand, the repetition enhancement subpopulation – originally interpreted in terms of these voxels being populated by prediction units – might reflect positive effects of stimulus repetition as the expected precision of (or confidence in) prediction errors increases with perceptual learning. As a result, even minimal differences between the descending predictions and the incoming sensory inputs will be amplified by strong precision weighting (for a more detailed discussion of the relation between confidence and precision, see e.g., FitzGerald et al., 2015, Kanai et al., 2015, Moran et al., 2013). The relatively late latency of repetition enhancement effects on the amplitude of the evoked response (Recasens et al., 2014) speaks exactly to the sort of slow neuromodulatory mechanisms (i.e., short-term plasticity) of perceptual learning discussed above. In summary, we again see the opposing effects of learning that mediates repetition suppression (of prediction errors) and the increase in precision or confidence in prediction errors with repetition that may underlie repetition enhancement (through precision weighting).

### 5.4 Oscillatory mechanisms of repetition suppression

As discussed in previous sections, the predictive coding framework postulates different spectral profiles of descending predictions and ascending prediction errors (Bastos et al., 2012). Since prediction errors are linearly accumulated over time in order to inform predictions, the implicit transformation of prediction errors into predictions will entail a loss of high frequencies. Conversely, the non-linear dependency of prediction errors on prediction augments high-frequency message passing from deep to superficial cortical layers. How do these spectral asymmetries hold in the context of repetition suppression? MEG studies in humans have shown suppression of gamma-band synchrony (as well as evoked responses) in auditory cortex following an expected stimulus repetition, consistent with a successful minimisation of the prediction error (Todorovic, van Ede, Maris, & de Lange, 2011). In the same study, unexpected stimulus repetitions were associated with stronger gamma-band responses than expected stimulus repetitions, indicating stronger prediction error signalling – possibly in superficial layers of auditory areas, although a direct assignment of prediction error propagation to a specific laminar profile remains to be established using intralaminar recordings – when the descending prediction did not match sensory input.

Gamma-band synchronisation in response to stimulus repetition has also been studied invasively in the macaque visual cortex (Brunet et al., 2014). Here, a repeated presentation of visual gratings increased visually-induced gamma-band activity in the primary visual cortex and in area V4, as well as gamma-band coherence between V1 and V4 activity. Although these findings might seem inconsistent with the results from the human auditory cortex, in which expected stimulus repetition is linked to decreased gamma-band activity, the macaque study also reported dissociations in the trial-by-trial evolution of gamma-band response depending on cell population. Interestingly, the repetition-dependent increase of gamma-band activity was limited to narrow-spiking cells in area V4 (putative inhibitory interneurons), while broad-spiking cells (putative pyramidal cells) showed repetition-dependent decrease of gamma-band activity, consistent with their role in signalling prediction error. The positive effect of stimulus repetition on inhibitory interneurons, on the other hand, supports their role in modulating pyramidal activity by providing a gain mechanism which can be described in terms of precision of prediction errors (cf. Auksztulewicz & Friston, 2015).

## 6 Predictive coding and alternative accounts of repetition suppression

One of the initially counterintuitive features or repetition suppression effects is that less neural activity is associated with improved behavioural performance. This apparent opposition has attracted various explanations in terms of facilitation, sharpening, and synchronisation (Gotts, Chow, & Martin, 2012). According to the “facilitation” hypothesis of repetition suppression (Henson, 2003, James and Gauthier, 2006, James et al., 2000), repeated stimuli evoke earlier and less prolonged neural activity than novel stimuli. In fMRI studies, the earlier onset of neural activity would not be detectable due to the slow time course of the BOLD response, while the shorter duration of evoked activity would be detectable as repetition suppression. The “sharpening” hypothesis (Desimone, 1996, Wiggs and Martin, 1998), on the other hand, postulates that repeated stimuli evoke on average less neural activity, although this decrease can largely be associated with cells that are poorly tuned to the stimulus. Conversely, the cells that are well-tuned to the stimulus are assumed to increase their response rate with stimulus repetition. Finally, the “synchronisation” model postulates that while repetition leads to lower overall firing rates, the cells responding to the stimulus fire more synchronously with one another, which should lead to more efficient neural processing (Gilbert, Gotts, Carver, & Martin, 2010).

The “facilitation” and “sharpening” phenomena are readily explained within the predictive coding framework. Facilitation, or a speeding of evoked neuronal responses, is equivalent to an in increase in synaptic rate constants. This is formally identical to an increase in synaptic gain, encoding the precision of prediction errors. Similarly, increased precision will boost prediction errors that will inform higher levels of the hierarchy about the most likely cause of sensory input, while suppressing other explanations of the sensorium. This is identical to sharpening of the neural representation of a given stimulus. Stimulus repetition will lead to monotonic changes in connections between cortical areas, enabling a more efficient (facilitated and sharpened) neural response to repeated stimuli.

The connections between cortical areas are subject to modulation of post-synaptic gain to specific inputs. Synchronisation of pre-synaptic inputs has been proposed as a likely candidate for controlling post-synaptic gain (Chawla, Lumer, & Friston, 1999). As discussed above, synchronisation of pre-synaptic inputs would show spectral asymmetries depending on the directionality of effective connectivity, with ascending connections being mediated by higher frequency bands than descending connections (Bastos et al., 2015a, Bastos et al., 2015b, Bosman et al., 2012, Buffalo et al., 2011, Roberts et al., 2013, Xing et al., 2012).

Similarly, several competing hypotheses had previously been proposed to account for a phenomenon closely related to repetition suppression, namely the mismatch negativity. According to the model-adjustment hypothesis (Näätänen and Winkler, 1999, Winkler et al., 1996), the MMN is a reflection of modifications of a perceptual model, occurring when the sensory input does not match the predictions of the model. The adaptation hypothesis (Jääskalainen et al., 2004, May et al., 1999), on the other hand, postulates that the MMN reflects adaptive changes in post-synaptic sensitivity during learning. As suggested by the DCM study of the roving oddball paradigm (Garrido, Kilner, Kiebel et al., 2009), the predictive coding framework accommodates both these accounts. In the context of repetition suppression, after each repetition of a stimulus, the perceptual model is optimised by increasing the precision-weighting of prediction errors. This entails a gradual increase in post-synaptic sensitivity to sensory inputs over the course of a sequence of standard stimuli.

Crucially, beyond accommodating previous, more particular accounts of repetition suppression, predictive coding – by postulating a central role for descending connections in perceptual inference and learning in conveying predictions of sensory inputs and their precision – readily explains contextual effects of attention, expectancy, and confidence on neural responses to repeated stimuli. At the same time, it generates testable hypotheses in terms of laminar specificity of prediction and prediction error propagation, as well as the key involved neurotransmitters and neuromodulators, whose interactions will be reflected in the amplitude and dynamics of observable repetition effects.

## 7 Conclusions

The predictive coding framework offers a mechanistic explanation of repetition suppression in terms of optimised predictions about the content and precision of sensory inputs. This dual role of predictions, mediated by descending connections between levels of the cortical hierarchy, explains how contextual factors such as attention, contextual expectancy, and prior experience might modulate the effect of stimulus repetition – by simultaneously minimising prediction errors in perceptual inference, and increasing their precision due to perceptual learning. The precision-modulation of the underlying prediction error suppression is likely due to NMDA-receptor dependent plasticity and classical neuromodulators such as ACh. The ensuing modulation of inhibitory populations in sensory regions will influence neural activity in superficial layers that are populated by neurons encoding prediction errors. By bridging the neurophysiological and computational levels of description, the predictive coding framework reconciles several accounts of repetition suppression and offers insights into the most general principles of neuronal message passing.
